# Synergistic acceleration of experimental tooth movement by supplementary high-frequency vibration applied with a static force in rats

**DOI:** 10.1038/s41598-017-13541-7

**Published:** 2017-10-25

**Authors:** Teruko Takano-Yamamoto, Kiyo Sasaki, Goudarzi Fatemeh, Tomohiro Fukunaga, Masahiro Seiryu, Takayoshi Daimaruya, Nobuo Takeshita, Hiroshi Kamioka, Taiji Adachi, Hiroto Ida, Atsushi Mayama

**Affiliations:** 1 Division of Orthodontics and Dentofacial Orthopedics, Graduate School of Dentistry, Tohoku University, Miyagi, 980-8575 Japan; 2 Department of Biomaterials and Bioengineering, Faculty of Dental Medicine, Hokkaido University, Hokkaido, 060-8586 Japan; 3 Department of Orthodontics, Graduate School of Medicine, Dentistry and Pharmaceutical Sciences, Okayama University, Okayama, 700-8525 Japan; 4 Department of Biomechanics, Research Center for Nano Medical Engineering, Institute for Frontier Medical Sciences, Kyoto University, Kyoto, 606-8507 Japan

## Abstract

Several recent prospective clinical trials have investigated the effect of supplementary vibration applied with fixed appliances in an attempt to accelerate tooth movement and shorten the duration of orthodontic treatment. Among them, some studies reported an increase in the rate of tooth movement, but others did not. This technique is still controversial, and the underlying cellular and molecular mechanisms remain unclear. In the present study, we developed a new vibration device for a tooth movement model in rats, and investigated the efficacy and safety of the device when used with fixed appliances. The most effective level of supplementary vibration to accelerate tooth movement stimulated by a continuous static force was 3 gf at 70 Hz for 3 minutes once a week. Furthermore, at this optimum-magnitude, high-frequency vibration could synergistically enhance osteoclastogenesis and osteoclast function via NF-κB activation, leading to alveolar bone resorption and finally, accelerated tooth movement, but only when a static force was continuously applied to the teeth. These findings contribute to a better understanding of the mechanism by which optimum-magnitude high-frequency vibration accelerates tooth movement, and may lead to novel approaches for the safe and effective treatment of malocclusion.

## Introduction

Orthodontic treatment can enhance the quality of life of patients with malocclusion by improving aesthetics, stomatognathic function, and psychological disorders. However, orthodontic treatment is often of long duration, is accompanied by risks such as dental caries and periodontal disease, and induces pain, discomfort^1,2^ and root resorption^3–5^. Attempts to accelerate tooth movement to shorten the duration of treatment by stimulating the remodelling activity of alveolar bone have included physical approaches (such as low-energy laser irradiation^6^, low intensity pulsed ultrasound^7^, vibration^8–10^ and pulsed electromagnetic fields^11^) and pharmaceutical approaches (such as local injection of prostaglandin E_2_^12^, 1,25-dihydroxyvitamin D_3_^13–15^ and parathyroid hormone^16^). However, adverse side effects such as local pain, discomfort and severe root resorption^17^ have been associated with these techniques.

Low-magnitude (LM; less than 1 g, where g = 9.81 m/s^2^) high-frequency (HF; 20–90 Hz) vibrations, such a mechanical signal, can positively influence skeletal homeostasis and stimulate an anabolic response in both weight-bearing^18^ and non-weight-bearing^19^ bone, and furthermore, in adult rats with ovariectomy-induced osteoporosis^20^. LMHF vibration is currently used as a safe and non-invasive treatment for bone loss in postmenopausal women^21^ and also to promote osteogenesis in children with disabling conditions^22^. In dental practice, several prospective randomised controlled clinical trials have recently investigated the effect on orthodontic tooth movement of supplemental vibration applied with fixed appliances for 20 min/day using a vibration device which delivers a force of 0.25 N (25.49 g) at a frequency of 30 Hz to the dentition^8–10^. Although some of these studies reported an increase in the rate of tooth movement when vibration was applied as an adjunct to orthodontic treatment^8^, others demonstrated that supplemental vibration did not increase the rate of tooth movement^9,10^. The difference in these treatment outcomes is controversial and still unclear. Previous studies have not investigated which vibration characteristics such as force magnitude and frequency and what exposure time and timing are the most effective and efficient without producing adverse side effects. Furthermore, the underlying cellular and molecular basis of this phenomenon *in vivo* remains unclear, although vibrational effects on mesenchymal stromal cells^23^ and osteoblasts^24^ have been investigated *in vitro*.

Bone is a dynamic organ subjected to a variety of mechanical loads during daily activity, and it has the capacity to adapt structurally by changing its mass, architecture, morphology and density in response to mechanical loading^25,26^ through the process of bone resorption and formation. Thus, bone cells are sensitive to their environment, and can detect chemical and mechanical signals^25–28^. Orthodontic force application (i.e. mechanical loading to teeth) is known to increase bone modelling and remodelling activity in both formation and resorption areas of alveolar bone in mice^29,30^, rats^31–33^ and dogs^34^. Therefore, acceleration of orthodontic tooth movement requires the stimulation of both bone resorption by osteoclasts on the compression side and bone formation by osteoblasts on the tension side. Osteocytes, which are buried in the bone, form cellular networks that enable communication not only with other osteocytes, but also with osteoclasts and osteoblasts on the bone surface in response to mechanical stress^35–37^. We have previously shown that osteocytes are mechano-sensing and play a vital role in osteoclastogenesis and bone resorption during orthodontic tooth movement^28,30,33,38,39^.

The transcription factor, nuclear factor-kappa B (NF-κB), is a pleiotropic mediator of stress-induced gene expression^40^. Signal transduction pathways of NF-κB as a mediator of cellular stress play a critical role in cell survival, growth and differentiation, apoptosis, and function in eukaryotic cells, including the major skeletal cell types such as osteoclasts, osteoblasts and osteocytes^41,42^. Mechanical stimulation is known to activate NF-κB signals in osteoblasts and related cells^42^ and, thereafter, influences bone metabolism as a result of cellular and molecular interactions in osteoclasts, osteoblasts and osteocytes. Therefore, we hypothesised that a dynamic vibration force applied with a continuous static force would exert synergistic effects to activate bone modelling and remodelling through osteoclasts, osteoblasts and osteocytes, resulting in acceleration of orthodontic tooth movement.

In the present study, we constructed a new vibration device for a tooth movement model in rats, and investigated the efficacy and safety of the vibration device applied with fixed appliances. Furthermore, to understand the underlying mechanism, we demonstrated for the first time that supplementary vibration applied with a continuous static force could enhance osteoclastogenesis and osteoclast function via NF-κB activation, leading to alveolar bone resorption, and consequently, accelerated orthodontic tooth movement.

## Results

### Determining the optimum-magnitude high-frequency vibration for accelerating tooth movement

**Figure 1:**
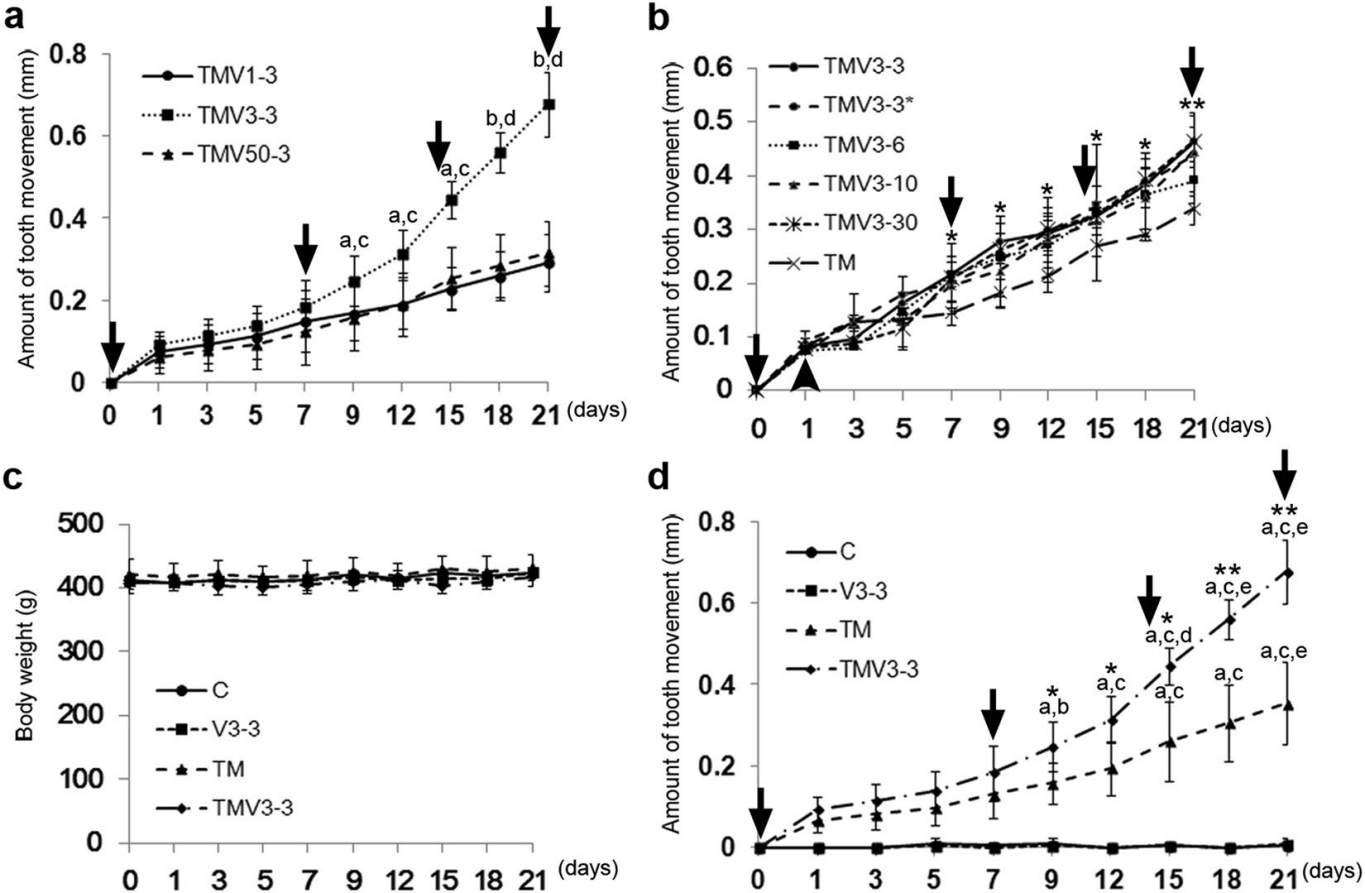
Effect of supplementary vibration applied with continuous static force on body weight and amount of tooth movement during the experimental tooth movement in rats. (**a**) Effect of various vibrations on the acceleration of tooth movement. One gf at 58 Hz (TMV1-3, n = 5), 3 gf at 70 Hz (TMV3-3, n = 4) or 50 gf at 278 Hz (TMV50-3, n = 4) of vibration force for 3 min were applied on days 0, 7, 14 and 21 after the initiation of experimental tooth movement (arrows). ^a^ *P* < 0.05 vs TMV1-3, ^b^ *P* < 0.01 vs TMV1-3, ^c^ *P* < 0.05 vs TMV50-3, ^d^ *P* < 0.01 vs TMV50-3. (**b**) Effect of the exposure duration of vibration on the acceleration of the experimental tooth movement. The amount of tooth movement by a continuous static force was measured (TM, n = 3). Supplementary vibration of 3 gf at 70 Hz was applied for 3 min (TMV3-3, n = 3), 6 min (TMV3-6, n = 2), 10 min (TMV3-10, n = 2) or 30 min (TMV3-30, n = 2) on days 0, 7, 14 and 21 of the experimental tooth movement by continuous static force (arrows). TMV3-3* (n = 2) indicated that supplementary vibration of 3 gf at 70 Hz was applied for 3 min on day 1 (arrowhead) instead of day 0, and the same timing thereafter. *P < 0.05 vs TM, **P < 0.01 vs TM. (**c**) Changes of body weight during experimental tooth movement of C, V3-3, TM, and TMV3-3. There was no significant difference in body weight among four experimental groups during the experimental period. (**d**) Time-course change in the amount of tooth movement of C, V3-3, TM and TMV3-3 groups. Arrows indicated the exposure of vibration of 3 gf at 70 Hz for 3 minutes. C (n = 2), control; V3-3 (n = 2), vibration of 3 gf at 70 Hz for 3 min; TM (n = 4), tooth movement by continuous static force; TMV3-3 (n = 4), supplementary vibration of 3 gf at 70 Hz for 3 min applied with a continuous static force. ^a^ *P* < 0.01 vs Day 1, ^b^ *P* < 0.05 vs Day 3, ^c^ *P* < 0.01 vs Day 3, ^d^ *P* < 0.05 vs Day 12, ^e^ *P* < 0.01 vs Day 12, *P < 0.05 vs TM, **P < 0.01 vs TM.

Several magnitudes of vibratory force, with a continuous static force generated by bent 0.014 inch nickel-titanium (Ni-Ti) wires, were applied to the rat maxillary right first molar to move it palatally. To assess the effects of various vibration forces on the experimental tooth movement, vibration forces of 1 gf at 58 Hz (TMV1-3), 3 gf at 70 Hz (TMV3-3) or 50 gf at 268 Hz (TMV50-3) were applied for 3 min at 1-week intervals over 21 days (Fig. 1a). The TMV3-3 group demonstrated significantly increased tooth movement compared with TMV1-3 and TMV50-3 groups on day 9 and thereafter; tooth movement was similar in the TMV1-3 and TMV50-3 groups (Fig. 1a). Thus, a force of 3 gf at a frequency of 70 Hz was determined to be the optimum vibration for accelerating tooth movement in the present study.

To evaluate the effects of various durations of exposure to vibration on the rate of tooth movement, vibration of 3 gf at 70 Hz was applied weekly for 3 min (TMV3-3), 6 min (TMV3-6), 10 min (TMV3-10), or 30 min (TMV3-30). The amount of tooth movement of each TMV group was significantly larger than that of the TM group. There was no significant difference in the amount of tooth movement among the TMV groups receiving supplemental vibration between 3 and 30 min once a week (Fig. 1b). Thus, because acceleration of experimental tooth movement by supplemental vibration did not correlate with the duration of the exposure to vibration, we chose a 3-minute application in the following experiments for efficiency and safety reasons.

The periodontal ligament (PDL) is compressed instantaneously on day 1^31^. To determine the timing of application of the supplemental vibration, vibration of 3 gf at 70 Hz was applied for 3 min on day 0 or day 1 after the initiation of tooth movement (Fig. 1b). There was no significant difference in the amount of tooth movement between the day 0 and day 1 groups. Therefore, in subsequent experiments, the supplemental vibration was applied to rat teeth at the time of the static force application.

Based on these results, we determined that the optimal conditions for effective and efficient supplemental vibration for accelerating tooth movement were 3 gf at 70 Hz for 3 min at 1-week intervals.

### Optimum-magnitude high-frequency vibration applied with static continuous force accelerates tooth movement

We measured the body weight of the rats daily for the 21 days of the experiments. The body weights of C (control; no vibration and no static force), V3-3 (vibration; 3 gf at 70 Hz for 3 min), TM (tooth movement by a continuous static force), and TMV3-3 groups were almost constant at approximately 410 g with no significant difference among these groups during the experimental period (Fig. 1c).

Figure 1d shows changes in the experimental tooth movement over time. On day 1 after the commencement of the experiments, rapid tooth movement of approximately 0.1 mm took place in the TM and TMV3-3 groups. The amount of tooth movement in the TM group increased further after day 1, and the increase on day 15 was significant when compared with that on day 1 and day 3 (Fig. 1d). In the TMV3-3 group, the amount of tooth movement on day 9, 48 h after the second vibration of 3 min on day 7, was significantly greater than that on both day 1 and day 3. Consequently, on day 9, the amount of tooth movement of the TMV3-3 group was significantly greater than that of the TM group. In addition, the amount of tooth movement in the TMV3-3 group significantly increased between day 12 and day 15, whereas there was no significant difference in the TM group. The TMV3-3 group exhibited twice the amount of tooth movement as the TM group at the end of the experiment on day 21. Vibration alone did not cause the tooth movement. Therefore, vibration did not produce a directional force that moved the teeth.

### Optimum-magnitude high-frequency vibration applied with static continuous force does not affect root resorption

**Figure 2:**
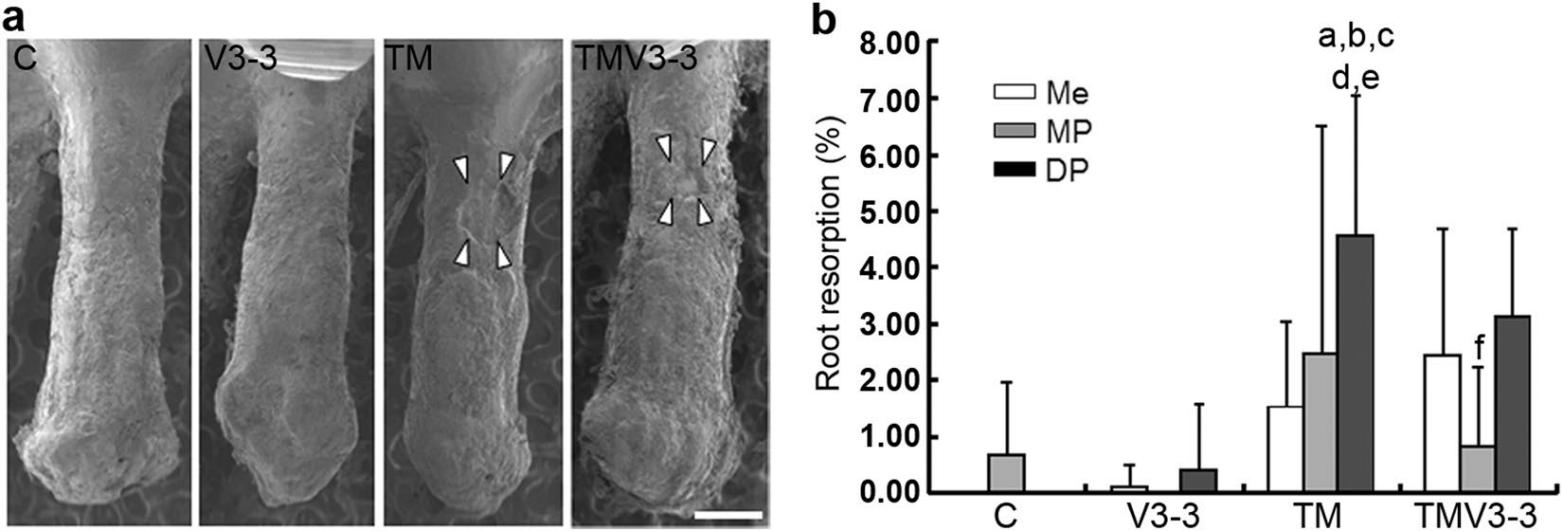
Effect of supplementary vibration applied with continuous static force on root resorption during the experimental tooth movement in rats. (**a**) SEM images of mesio-palatal root of maxillary 1^st^ molar on day 21 of C, V3-3, TM and TMV3-3 groups. The area surrounded by white arrowheads indicated root resorption lacuna. (**b**) Evaluation of the ratio of root resorption areas in mesial, mesio-palatal, and disto-palatal roots of C, V3-3, TM and TMV3-3 groups (all groups, n = 4) on day 21. Mesial and disto-palatal roots of C group and mesio-palatal root of V3-3 group were not detected resorption lacunae. Scale bar = 500 μm. DP, disto-palatal root; Me, mesial root; MP, mesio-palatal root. ^a^ *P* < 0.01 vs Me in C, ^b^ *P* < 0.01 vs DP in C, ^c^ *P* < 0.01 vs Me in V3-3, ^d^ *P* < 0.01 vs MP in V3-3, ^e^ *P* < 0.01 vs DP in V3-3, ^f^ *P* < 0.05 vs DP in TM.

To determine the effect of supplemental vibration on root resorption, we measured the areas of root resorption on scanning electron microscope (SEM) images of the area of compression on day 21. In the C and V3-3 groups, the root surface was covered with smooth cementum, and root resorption lacunae were scarcely observed (Fig. 2a). In the TM and TMV3-3 groups, root resorption lacunae appeared mainly around the furcation area of the roots (Fig. 2a). The amount of root resorption of the mesial, mesiopalatal, and distopalatal roots did not differ between the C and V3-3 groups, and accounted for less than 1% of the root surface (Fig. 2b). The TM group showed increased root resorption in all mesial, mesiopalatal and distopalatal roots, and the resorption was significantly greater in the distopalatal root than the other roots in the C and V3-3 groups (Fig. 2b). The TMV3-3 group also showed a tendency for increased root resorption in the mesial and distopalatal roots, but not significant compared with the C and V3-3 groups (Fig. 2b). Furthermore, root resorption of the mesiopalatal root in the TMV3-3 group was significantly smaller than that of the distopalatal root in the TM group (Fig. 2b). The optimum-magnitude of high-frequency vibration for accelerating tooth movement did not exacerbate root resorption when compared with continuous static force alone.

### Optimum-magnitude high-frequency vibration applied with static continuous force induces NF-κB activation in osteoclasts, osteoblasts and osteocytes

**Figure 3:**
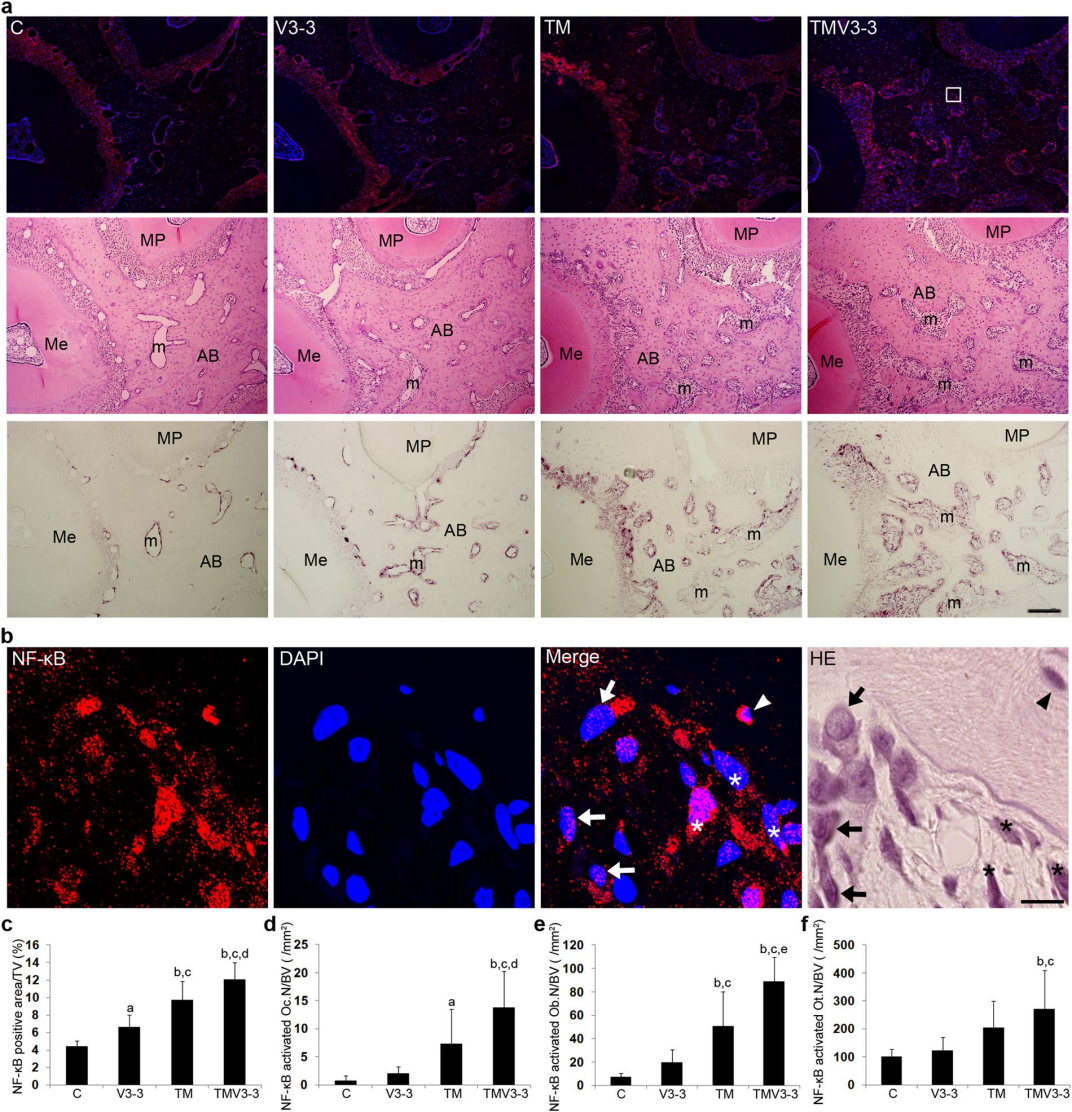
Effects of supplementary vibration applied with continuous static force on NF-κB p65 activation in the osteoclasts, osteoblasts and osteocytes on day 9 of the experimental tooth movement. (**a**) Horizontal sections of upper 1^st^ molar were obtained from C, V3-3, TM, and TMV3-3 groups on day 9. Serial sections were treated with anti- NF-κB p65 antibody, and stained with HE and TRAP. A rectangle indicated the region of magnification shown in panel b. AB, alveolar bone; m, bone marrow; Me, mesial root; MP, mesio-palatal root. Scale bar = 100 μm. (**b**) TMV3-3 group increased nuclear translocation of NF-κB p65 in osteoclasts, osteoblasts, and osteocytes. *, oeteoclasts translocated NF-κB in their nucleus; arrow, osteoblasts translocated NF-κB in their nucleus; arrowhead, osteocytes translocated NF-κB in their nucleus. Scale bar = 10 μm. (**c**,**d**,**e**,**f**) Histomorphometric analysis of the area of NF-κB expression to total volume (**c**), the number of osteoclasts translocated NF-κB in their nucleus to bone volume (**d**), the number of osteoblasts translocated NF-κB in their nucleus to bone volume (**e**), the number of osteocytes translocated NF-κB in their nucleus to bone volume (**f**). C (n = 3), control; V3-3 (n = 3), vibration of 3 gf at 70 Hz for 3 min; TM (n = 3), tooth movement by continuous static force; TMV3-3 (n = 3), supplementary vibration of 3 gf at 70 Hz for 3 min applied with continuous static force. ^a^ *P* < 0.05 vs C, ^b^ *P* < 0.01 vs C, ^c^ *P* < 0.01 vs V3-3, ^d^ *P* < 0.05 vs TM, ^e^ *P* < 0.01 vs TM.

NF-κB plays an important role in the adaptive response to physiological stimuli including mechanical stress^40,42^. Therefore, we performed immunofluorescence to examine the effects of supplementary vibration on NF-κB activation in osteoclasts, osteoblasts and osteocytes during experimental tooth movement. The expression of NF-κB p65 in the cytoplasm and nuclei was significantly greater in the V3-3, TM and TMV3-3 groups than C group on day 9, 48 h after the second application of vibration for 3 min on day 7 of the experiment (Fig. 3a and c). The TM group exhibited a greater increase in NF-κB expression than the V3-3 group (Fig. 3c). The most intense expression of NF-κB was observed in the TMV3-3 group (Fig. 3c).

Next, we investigated NF-κB activation in osteoclasts, osteoblasts and osteocytes. The cells expressing NF-κB p65 in the nuclei were recognised as activated NF-κB-positive cells^40–42^. In addition, we stained serial sections with tartrate-resistant acid phosphatase (TRAP) and haematoxylin and eosin (HE) staining for identification of osteoclasts, osteoblasts and osteocytes (Fig. 3a and b). There were several NF-κB-activated osteoclasts and osteoblasts in the C group. The V3-3 group showed no increased activation of NF-κB in osteoclasts, osteoblasts or osteocytes (Fig. 3a,d,e and f). The TM and TMV3-3 groups had a significantly greater number of NF-κB-activated osteoclasts and osteoblasts compared with the C and V3-3 groups (Fig. 3a,d and e). The largest number of activated NF-κB-positive osteoclasts, osteoblasts and osteocytes were observed in the TMV3-3 group (Fig. 3a,b,d,e and f). The TM group tended to have more NF-κB-activated osteocytes than the C and V3-3 groups (Fig. 3f). Thus, supplementary vibration applied with a static continuous force synergistically stimulated the activation of NF-κB in osteoclasts, osteoblasts and osteocytes.

### Optimum-magnitude high-frequency vibration applied with static continuous force increases periodontal ligament (PDL) volume via osteoclastic bone resorption

**Figure 4:**
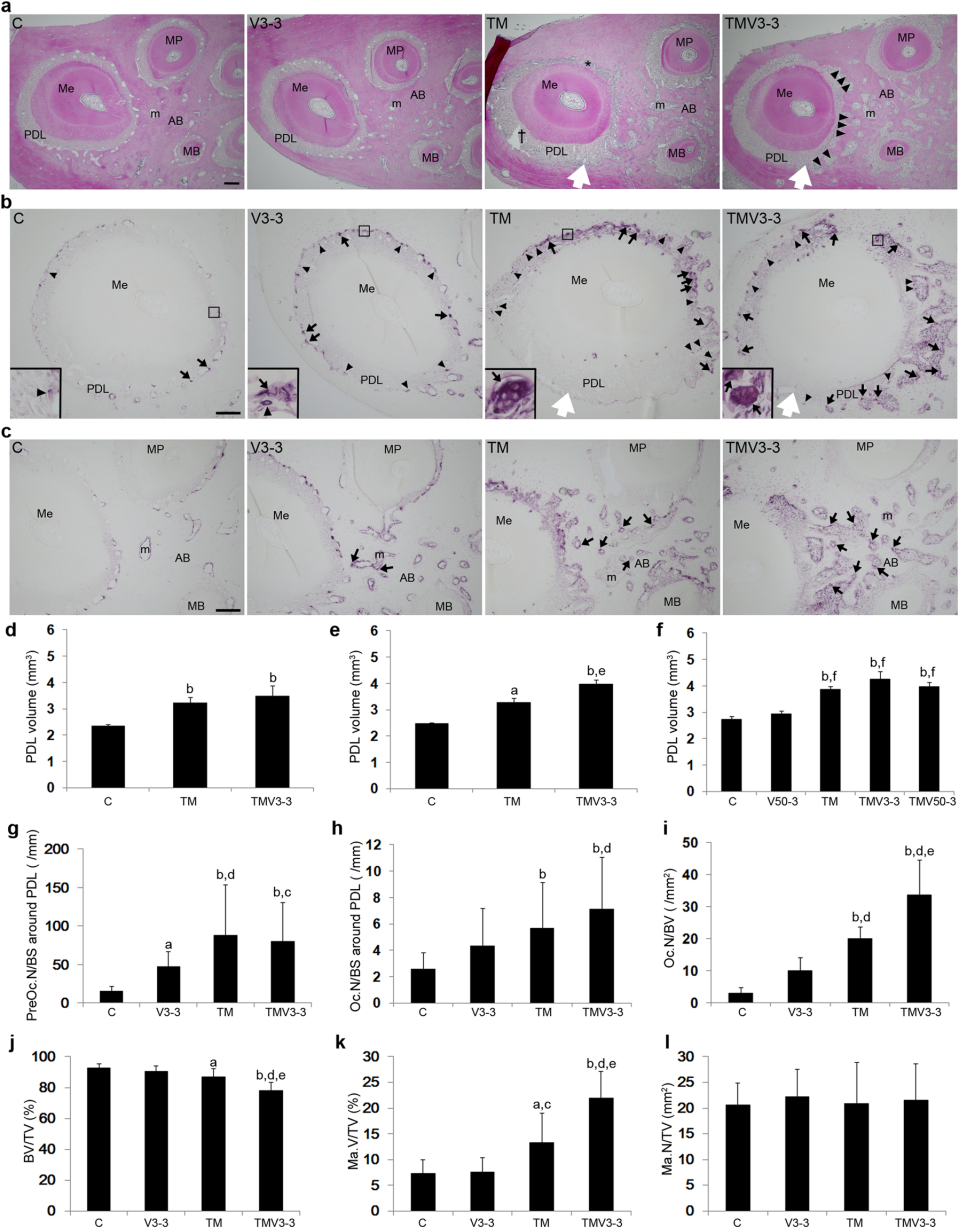
Effect of supplementary vibration applied with a continuous static force on the PDL and the bone marrow. (**a**) Histological observation by HE staining of upper 1^st^ molar obtained from C, V3-3, TM, and TMV3-3 groups on day 9 of the experimental tooth movement. PDL was compressed (*) in the compression side and stretched (†) in the tension side in TM group. In TMV3-3 group, the width of PDL in the compression side increased with formation of irregular bone resorption surfaces (arrowheads). White arrow indicates the direction of a static force. (**b**) TRAP-positive cells in PDL of the mesial root of the maxillary 1^st^ molar. TRAP-positive cells were rarely observed in C group. Preosteoclasts (arrowheads) and osteoclasts (arrows) appeared on the surface of alveolar bone all around the PDL in V3-3 group. TM group increased osteoclasts in the compression side. TMV3-3 group increased osteoclasts in both tension and compression sides all around PDL. Rectangles in each section indicated the areas enlarged in the inset. (**c**) TRAP-positive cells in deep alveolar bone. Osteoclasts (arrows) were rarely observed in deep alveolar bone in C group but appeared in V3-3 group. TM and TMV3-3 groups increased osteoclasts in deep alveolar bone. AB, alveolar bone; MB, mesio-buccal root; Me, mesial root; MP, mesio-palatal root; m, bone marrow; PDL, periodontal ligament; white arrow, directions of tooth movement. Scale bar = 200 μm. (**d**,**e**,**f**) µCT evaluation of the PDL volume of the maxillary 1^st^ molar on day 7 (**d**), day 9 (**e**), and day 21 (**f**) in C, V3-3, V50-3, TM, TMV3-3 and TMV50-3 groups (all groups, n = 4). (**g**,**h**) Histomorphometric analysis of number of preosteoclasts (**g**) and osteoclasts (**h**) on bone surface facing PDL of the mesial root in C, V3-3, TM and TMV3-3 groups (all groups, n = 3). (**i**,**j**,**k**,**l**) Histomorphometric analysis of number of osteoclasts to bone volume (Oc.N/BV), bone volume to tissue volume (BV/TV), marrow volume to tissue volume (Ma.V/TV), and marrow number to tissue volume (Ma.N/TV) in ROI of deep alveolar bone in C, V3-3, TM and TMV3-3 groups (all groups, n = 3). ^a^ *P* < 0.05 vs C, ^b^ *P* < 0.01.

The PDL in the TM group was compressed on the compression side and stretched on the tension side, although the width of the PDL was almost constant in the C and V3-3 groups (Fig. 4a). The width of the PDL in the TMV3-3 group was greater where irregular resorption had occurred on the bone surface of the compression side (Fig. 4a).

To determine the effects of supplemental vibration on bone resorption around the roots, we assessed the PDL volume around the maxillary first molar by µCT analysis. On days 7, 9 and 21, the PDL volumes of the TM and TMV3-3 groups were significantly greater than that of the C group. There was no significant difference in PDL volume between the TM and TMV3-3 groups on days 7 and 21 (Fig. 4d and f). When tooth movement was significantly accelerated by supplemental vibration on day 9, 48 h after the second vibration application for 3 min on day 7, the PDL volume of the TMV3-3 group was significantly greater than that of the TM group (Fig. 4e).

Next, we investigated the number of TRAP-positive cells on the bone surfaces around the PDL on day 9 (Fig. 4b) The V3-3 group had significantly more preosteoclasts along the bone surface around the PDL compared with the C group and, although there were also more osteoclasts around the PDL, this increase was not significant (Fig. 4g and h). The TM group had significantly more TRAP-positive preosteoclasts and osteoclasts on the compression side of the PDL on day 9 compared with the C group (Fig. 4b,g and h). There were significantly more osteoclasts on the compression and tension sides in the TMV3-3 group than in the TM group on day 9 (Fig. 4b and h). These results indicate that during the experimental tooth movement, vibration and a static continuous force increased the size of the tooth socket around the PDL by osteoclastic bone resorption.

### Optimum-magnitude high-frequency vibration applied with static continuous force promotes bone resorption in deep alveolar bone during tooth movement

We performed histomorphometric analysis to determine the effect of vibration on deep alveolar bone during the experimental tooth movement on day 9. Osteoclasts were only sparsely observed deep in the alveolar bone in the C group (Fig. 4c and i). The V3-3 group tended to have more osteoclasts than the C group, but the difference was not significant. Significantly more osteoclasts were observed in the TM and TMV3-3 groups than in the C and V3-3 groups. The TMV3-3 group synergistically increased the number of osteoclasts compared with the TM group (Fig. 4c and i). The TM and TMV3-3 groups exhibited a significant decrease in bone volume compared with the C and V3-3 groups and TMV3-3 group was significantly decreased more than TM group (Fig. 4j). This decreased bone volume in the TM and TMV3-3 groups was accompanied by an increase in the volume of the bone marrow, but not in the number of the bone marrow (Fig. 4k and l). There was no significant difference of an osteocyte lacunar area among C (mean ± S.D., 49.2 ± 11.5 μm^2^), V3-3 (48.5 ± 8.9 μm^2^), TM (50.1 ± 12.8 μm^2^) and TMV3-3 (53.1 ± 11.6 μm^2^) groups.

## Discussion

In this study, we demonstrated definitively that the application of optimum-magnitude high-frequency vibration with a continuous static force results in acceleration of tooth movement without root resorption in rats. We fabricated an orthodontic appliance for rats from bent Ni-Ti wire and produced a light continuous force of 15 gf on the maxillary first molar to simulate the application of orthodontic tooth movement in humans. We constructed a small and compact vibration generator and motor which produces optimum magnitude and high frequency vibration for orthodontic tooth movement, and is easy to apply not only to rats but also to humans in the clinic. This vibration device allows the vibration force to be controlled in clinical situations, with adjustments from 0.5 gf to 73 gf, and with automatic changes in the frequency of vibration from 48.3 Hz to 284.1 Hz. Using this vibration device, we investigated the possibility of accelerating orthodontic tooth movement by supplementary application of the vibration device, searching for the smallest number of applications, the smallest magnitude and the shortest exposure. We determined that the most effective magnitude of vibration to accelerate orthodontic tooth movement of the maxillary molar of adult rats was 3 gf at 70 Hz for 3 min per week.

Considering future clinical application of the vibration device in adult patients, we used 25-week-old male Wistar rats, which are considered as adults. Adult rats exhibit no skeletal growth, a lower rate of bone turnover, and slower orthodontic tooth movement than young rats^14,43^. These biological features of adult rats facilitate reproducible evaluation of the precise increase in the rate of tooth movement by application of the vibration device. The rats exhibited no change in body weight regardless of whether the vibration was accompanied by static orthodontic force or not.

Bone is constantly remodelled by the balanced activities of bone resorption and formation, and these phases in humans last approximately 1 to 2 weeks and 2 to 3 months, respectively^44^. In rats, resorption lasts approximately 1.5 days and reversal approximately 3.5 days; the forming phase is about 1 day, and the total duration of each remodelling cycle of rat alveolar bone is approximately 6 days^31^. Because we intend to apply the vibration device to patients at each monthly visit, we applied the vibration to the rat maxillary molars once a week, based on the length of the remodelling cycle. Vibration with a force of 3 gf was most effective in accelerating orthodontic tooth movement in the rats, in the range from 1 gf to 50 gf. However, there were no significant differences among the various vibration exposure durations (3, 6, 10 and 30 min) in accelerating orthodontic tooth movement. Thus, the acceleration of orthodontic tooth movement by supplementary dynamic vibration applied with a continuous static force may depend on the magnitude of the vibration force rather than the exposure duration.

Orthodontic tooth movement by the application of continuous force is typically divided into three phases: (1) instantaneous tooth movement by compressive deformation of the PDL; (2) arrested or slow tooth movement marked by the appearance of necrosis of the PDL on the compression side; and (3) resumption of linear rapid tooth movement caused by osteoclastic bone resorption^31,45^. In this study using adult rats, the first phase was observed at day 1 of orthodontic tooth movement; the second phase was seen between day 1 and 9; and the third phase was observed until day 21, which was the end of the experiment. Supplementary dynamic vibration applied with continuous static force had little effect on acceleration of tooth movement during the first and second phases. However, after day 9, supplementary vibration applied with continuous static force caused significantly faster tooth movement than static force alone, and achieved an approximately 2-fold increase in the amount of tooth movement by static force alone on day 21. Dynamic vibration alone did not cause any orthodontic tooth movement or any directional movement for correcting malpositioned teeth, but did cause tooth oscillation within the PDL in the tooth socket.

The aim of accelerating orthodontic tooth movement is to shorten the treatment period. However, the vibration device should be applied cautiously in orthodontic patients, because it is important to avoid side effects such as root resorption, pain and discomfort. It has been reported that some degree of root resorption occurs in almost all patients during orthodontic treatment, ranging from minor to severe^46–48^. In animal models, it was demonstrated that experimental tooth movement by means of both light and heavy forces induced root resorption in mice^4^ and rats^49^. Therefore, we evaluated the effect of the vibration device on root resorption. Consistent with previous findings^4,49^, experimental tooth movement under a continuous static force of 15 gf induced root resorption in rats. Dynamic vibration applied with continuous static force also induced a certain degree of root resorption compared with the control group; however, the increase was not significant. Interestingly, the amount of root resorption in the TMV3-3 group tended to be less than that of the TM group, even though tooth movement of the TMV3-3 group was faster than that of the TM group. Furthermore, optimum magnitude high frequency vibration alone did not induce root resorption. Thus, regulated vibration might cause no iatrogenic damage to tooth roots, even when applied with a continuous static force for accelerating tooth movement.

Previous studies have not investigated whether dynamic vibration acts on the PDL in a different way from static force. When continuous static force is applied to teeth, the PDL around the teeth is compressed on one side and stretched on the opposite side, resulting in compression and tension sides in the alveolar bone, respectively^28–30^, indicating that continuous compressing and stretching forces have a single directional vector. In this study, we investigated the distribution of osteoclasts in the PDL response to mechanical force. Dynamic vibration induced preosteoclasts and osteoclasts along the alveolar bone surface all around the PDL. However, there were significantly more osteoclasts along the bone surface on the compression side of the PDL under a continuous static force. Moreover, supplementary dynamic vibration applied with a continuous static force synergistically increased osteoclast recruitment not only on the compression side, but also on the tension side around the PDL. Based on the dynamic model proposed by Noyes and Solt^50^, which is calculated using the parameters of each element, vibration of 0.03 N force at 100 Hz translates to a displacement of 0.04 μm in the human periodontal membrane. Thus, it is assumed that vibration induces a micro-level oscillatory movement of the teeth. Taken together, it is suggested that oscillatory dynamic vibration and one-directional continuous static force affect cells in the PDL differently, and may have a different mode of action.

All mammalian cells have components of a dimeric transcription factor (NF-κB) signalling pathway, which is able to differentially regulate the expression of a diverse array of genes in a cell- and stimulus-specific manner^40^. NF-κB plays a critical role in cell growth and differentiation, apoptosis, and the adaptive response to cellular stress including physiological stimuli such as mechanical stress^40^. In resting cells, the primary mode of NF-κB regulation is cytoplasmic localisation as a result of their interactions with IκBs, which mask nuclear localisation signals. To be active, NF-κB dimers must be translocated into the nucleus^41^.

Orthodontic force applied to teeth is transmitted initially to the PDL. In the present study, the application of dynamic vibration with continuous static force significantly increased NF-κB activation in osteoclasts, and the number of preosteoclasts and osteoclasts on the bone surface in the PDL. The volume of the PDL expanded significantly on day 7 and even more on day 9, and the experimental tooth movement accelerated markedly until day 21. These findings suggest that dynamic vibration when applied with static force could synergistically increase the number of osteoclasts expressing NF-κB and promote osteoclast activation by tooth oscillation, resulting in enhancement of bone resorption with PDL enlargement, finally followed by an increased amount of tooth movement. Additionally, all bone cells (osteoclasts, osteoblasts and osteocytes) responded not only to a continuous static force but also to dynamic vibration, which induced the activation of NF-κB. On day 9 of the experimental tooth movement, the tooth continued to move linearly during the third phase, and the rate of tooth movement in the TMV3-3 group was significantly higher than that of the TM group. During this period, the continuous static force significantly increased the nuclear translocation of NF-κB in osteoclasts, osteoblasts and osteocytes, and its nuclear translocation was further increased synergistically by applying supplementary vibration with the continuous static force. Osteoclast formation requires NF-κB activation in osteoclast precursors and NF-κB pathways is necessary for osteoclast differentiation and function, and bone resorption^41,51^. These findings suggested that active bone resorption was induced by supplementary vibration applied with a continuous static force via NF-κB signalling in osteoclasts. Furthermore, NF-κB has been reported to modulate the differentiation, function and activity of skeletal cell types; not only osteoclasts but also osteoblasts, osteocytes and chondrocytes^41^. Further investigation is needed to clarify the biological roles of the transcripts of NF-κB target genes in these cells to stimulate bone modelling and remodelling during orthodontic tooth movement.

Next, to investigate the role of stimulated nuclear translocation of NF-κB in osteoclasts located at the interface with the bone marrow (the endosteum) situated in the deep bone matrix remote from the PDL, we also analysed the number of osteoclasts there. On day 9 of the experimental tooth movement, continuous static force had increased the number of osteoclasts along the endosteum of the bone marrow located deep in the alveolar bone, and supplementary vibration applied with a continuous static force synergistically increased the number of osteoclasts, suggesting that bone resorption was enhanced via NF-κB activation in osteoclasts and other bone cells. Consequently, supplementary vibration with a static force significantly decreased bone volume in deep areas of the alveolar bone as a result of an increase in bone marrow volume, rather than from an increase in its number. Furthermore, vibration and/or a continuous static force did not enlarge osteocyte lacunar areas. Therefore, we demonstrated for the first time that supplementary vibration applied with static continuous force decreased bone volume through bone resorption by osteoclasts on the bone surface, and not directly by osteocytes situated deep in the bone matrix. This concept is known as “osteocytic osteolysis”^52^. Although this concept has been recognised as one of the osteocyte’s functions and is supported by various recent reports^53–55^, our results suggest that vibration accelerated tooth movement in a synergistic manner with static force via direct osteoclastic bone resorption.

**Figure 5:**
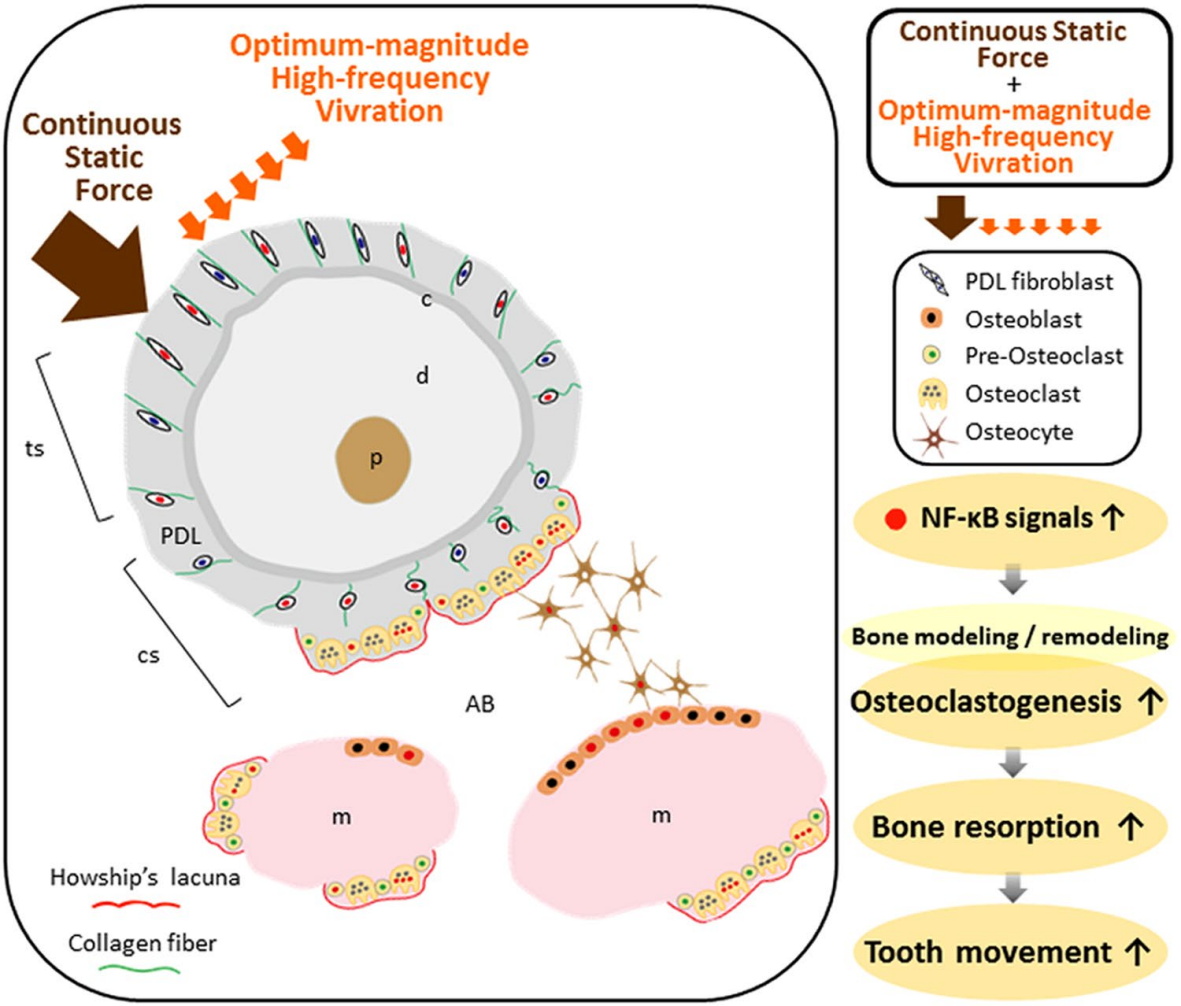
Model for the tooth movement stimulated by supplementary optimum-magnitude high-frequency vibration applied with a continuous static force. Supplementary high-frequency vibration applied with a continuous static force upregulates directly or indirectly NF-κB in PDL fibroblasts, osteoclasts, osteoblasts and osteocytes in PDL and deep alveolar bone around tooth root. NF-κB signals induced by such different mode of mechanical forces might play an important role to synergistically stimulate osteoclastogenesis and bone resorption, and accelerate tooth movement. AB, alveolar bone; c, cementum; cs, compression side; d, dentin; m, bone marrow; p, pulp; PDL, periodontal ligament; ts, tension side.

In conclusion, we developed a new vibration device for a tooth movement model in rats, and demonstrated for the first time that, during orthodontic tooth movement, optimum-magnitude high-frequency vibration could be directly transmitted to the PDL and bone cells, and enhance osteoclastogenesis and osteoclast function via NF-κB activation leading to alveolar bone resorption, and consequently accelerated tooth movement, but only when static force is continuously applied to the teeth (Fig. 5). Our data not only contribute to a better understanding of the mechanism by which tooth movement is accelerated by optimum-magnitude high-frequency vibration, but also suggest novel approaches for the safe and effective treatment of malocclusion. However, further study in clinical sciences is needed to fully elucidate the underlying mechanism.

## Methods

### Construction of a new vibration device

**Figure 6:**
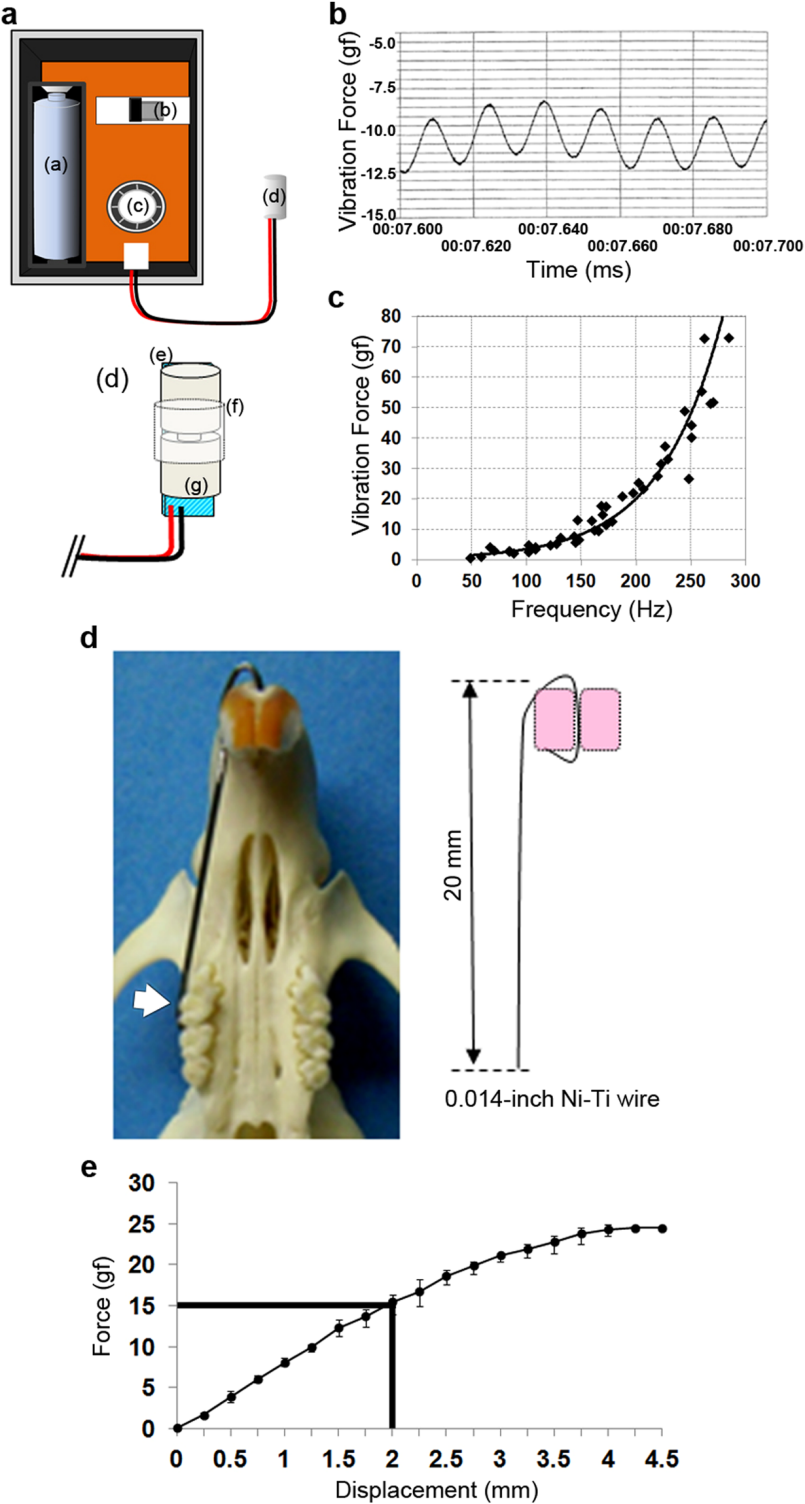
Characteristics of the constructed vibration generator and an appliance made by Ni-Ti wire for the experimental tooth movement in rats (**a**), Schematic drawing of vibration generator developed for this study was shown. (**a**) AAA size battery; (**b**) switch for generator; (**c**) variable resistance control; (**d**) vibration motor; (**e**) cylindrical ABS resin cover; (**f**) sheets of Ethylene-Vinyl Acetate (EVA) (1.5 mm) and polystyrene (1 mm); (**g**) 4 × 2 (mm/length, mm/diameter) vibration motor spindle. (**b**) Characteristics of the vibration. Vibration load was value of peak-to-peak force of acquired waveform from the vibration generator. (**c**), Force-frequency curve of the motor. In this study, we used the three kinds of vibration forces such as 1 gf at 58 Hz, 3 gf at 70 Hz, and 50 gf at 268 Hz. (**d**), Photograph and schematic drawing of an appliance made by Ni-Ti wire for the experimental tooth movement in the rat. White arrow indicates the direction of force applied by 0.014 inches Ni-Ti wire. (**e**) Force-displacement curve of the appliance. Four appliances were used to perform load-displacement measurement.

A high-frequency vibration generator with an eccentric weight at the tip of the rotation motor (an eccentric vibration motor) was newly constructed for this study and clinical use in the future (Fig. 6a). A vibration motor (KHN4NZ1X; Minebea Motor Manufacturing Corp., Tokyo, Japan) was connected with a lead wire to a battery box containing an AAA size battery. The battery box had a variable resistance control to alter the voltage input into the eccentric vibration motor. To prevent rotation of the vibration motor on contact with teeth and periodontal tissues, a cylindrical Acrylonitrile-Butadiene-Styrene (ABS) resin covered the motor. The characteristics of the constructed vibration generator are shown in Fig. 6b. A characteristic of the rotation motor was that the rotation speed, frequency, and force of vibration increased as the input voltage increased. Thus, the frequency and force could not be changed separately. The vibration device achieved multiple loading conditions of vibration (Fig. 6c). We used the vibration motor with a diameter of 2 mm and length of 4 mm for the application of peak-to-peak forces of 1 gf, 3 gf, or 50 gf, for which the frequency was automatically determined as 58 Hz, 70 Hz, or 268 Hz, respectively (Fig. 6c).

### Experimental tooth movement by using static orthodontic force and supplementary vibration

Bent Ni-Ti wire, a diameter of 0.014 inches and length of 20 mm, was fixed to the maxillary incisor by a composite resin for dental filling (UniFil LoFlo Plus; GC Co., Tokyo, Japan), and the maxillary right 1^st^ molar was moved palatally for 21 days, because of thinner alveolar bone on the buccal side (Fig. 6d). The force loaded to the molar was directly measured on the plaster model before and after placement of the wire in each rat, by a dial tension gauge (DTG-10NP; Mitutoyo, Kawasaki, Japan). When the wire tip was deflected 2 mm, loading was a 15 gf based on the load-displacement curve (Fig. 6e). A 15 gf orthodontic force was applied to the 1^st^ molar in this study. Although rat incisors were constantly erupting, once this appliance with loading had been placed, no further adjustment was necessary until 21 days.

Dynamic vibration was applied for teeth by placing the vibration motor in the palates of the rats to attach to bilateral 1^st^ molars. To fix the vibration motor in the palate, a ligature wire (ϕ 0.2 mm) was tied with 1^st^ and 2^nd^ molars bilaterally to vibration motor placed in the palate. Vibrating force magnitude and frequency were measured by strain gauge load cell (LTS-500GA; Kyowa Electronic Instruments Co., Ltd., Tokyo, Japan).

### Experimental animals

For experiments, 70 male 25-week-old Wistar rats weighing approximately 410 g were used. During the experiments, the rats were kept in cages in a room maintained at 21–24 °C with a 12-h/12-h ligt/dark cycle, and were fed a granular diet (Oriental Yeast, Tokyo, Japan) to prevent them from exerting an excessive chewing force.

The rats were divided randomly into two groups. In one group, 1^st^ molar on the left side was used as control (C group), and that on the right side was subjected to tooth movement by activated Ni-Ti appliance (TM group). In the other group, 1^st^ molar on the left side was subjected to vibration (V group) and that on the right side to tooth movement by activated Ni-Ti appliance and vibration (TMV group). All experimental protocols were approved by the Tohoku University of Science Animal Care and Use Committee. The care and handling of animals were performed in accordance with NIH guidelines.

### Measurement of tooth movement

Maxillary impressions of rats were taken with a silicon impression materials (Exafast injection type; GC Co.) under isoflurane inhalation anesthesia on days 0, 1, 3, 5, 7, 9, 12, 15, 18, and 21 during experimental tooth movement. Models of maxillary dentition were produced using super hard plaster. After trimming of the model was carried out, the occlusal surface view of the 1^st^, 2^nd^ and 3^rd^ molars were read with the scanner (GT-X970; SEICO EPSON Co., Tokyo, Japan). The conditions of the scan were set to 300dpi. Next, the picture was printed out in the size of ten times and traced out crowns, cusps, and palate folds. Finally, the trace was superimposed upon the trace before the tooth movement on the basis of 2^nd^ and 3^rd^ molars and measured the distance between the palatal cusps of the maxillary 1^st^ molars with digital calipers. A tenth of the distance was calculated as the amount of tooth movement. For each rat, the measurement was taken four times, and the mean value was used.

### Scanning electron microscope (SEM) observation and measurement of the root resorption area

**Figure 7:**
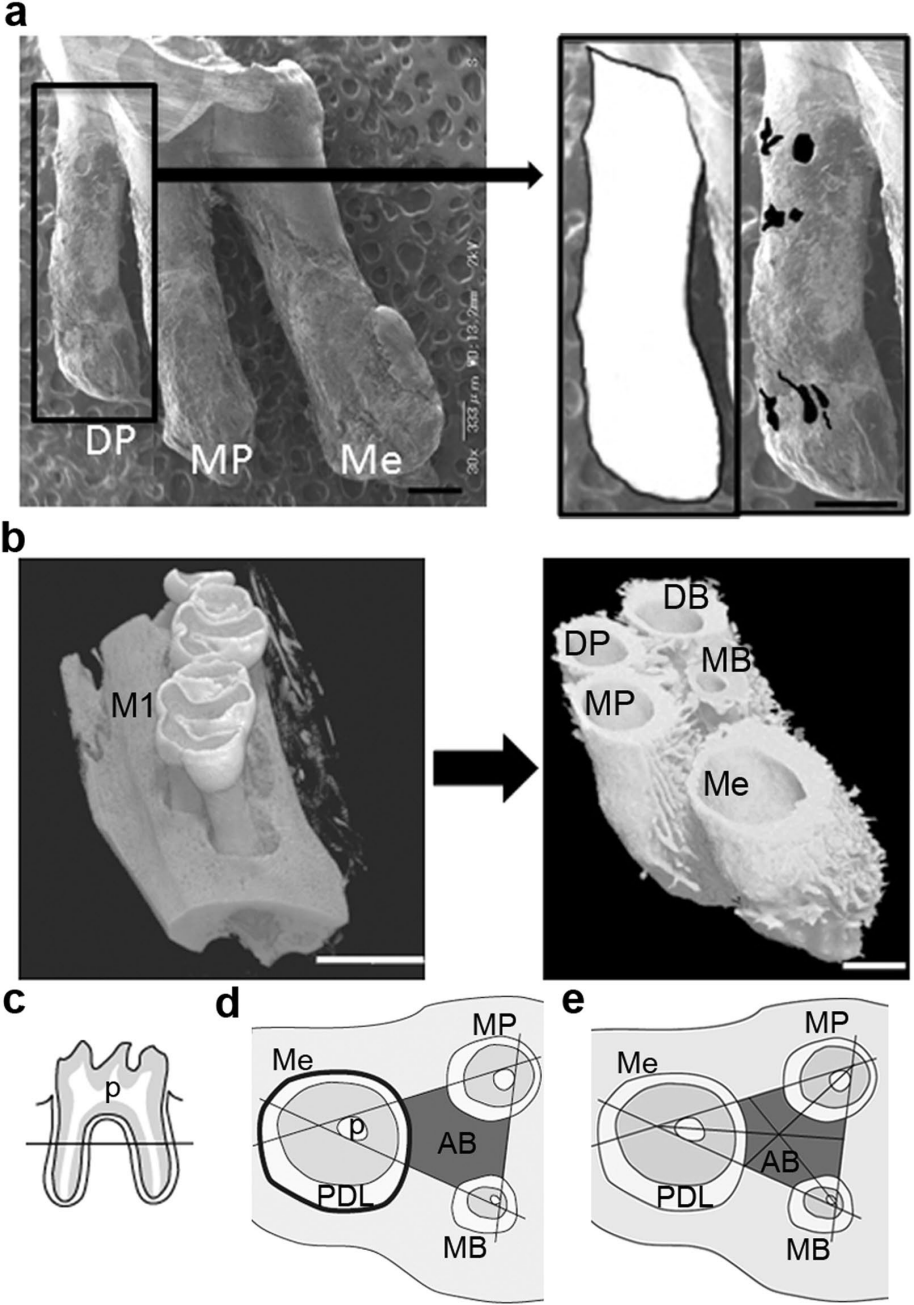
Histomorphometric analysis of root resorption, periodontal volume and alveolar bone. (**a**) Representative image of the mesial (Me), mesio-palatal (MP), and disto-palatal (DP) roots scanned by SEM on day 21 of the experimental tooth movement. Black box indicated the region of magnification shown in right panels. Right panels indicated root surface area (White) and resorption areas of the root (Black). The morphology of the root resorption lacunae varied round, oval, and irregular. Scale bar = 500μm. (**b**) Evaluation of the volume of the periodontal space of the maxillary 1^st^ molar of rats. Left panel showed representative µCT image of the maxilla after the experimental tooth movement. Right panel showed the reconstructed 3D image from µCT image of PDL and roots of the maxillary 1^st^ molar. Scale bar = 2 mm (left panel), 500 μm (right panel). (**c**) For histomorphometric analysis, first section of serial horizontal sections was obtained at 415 μm from the furcation of the maxillary 1^st^ molar. (Line). (**d**) A diagram indicated the area for histomorphometric analysis in a horizontal section of the maxillary 1^st^ molar, PDL and alveolar bone. The field of alveolar bone surrounded by lines tangential to each pulp surface of each root (dark gray) was measured for histomorphometric analysis (ROI). Bold line around Me indicated bone surfaces around the PDL of Me. (**e**) A diagram indicated the area for histomorphometric analysis of osseous lacunae. The size of osseous lacunae was measured in six fields divided by three median lines of the ROI’s triangle. AB, alveolar bone; DB, disto-buccal root; DP, disto-palatal root, M1; maxillary 1^st^ molar; MB, mesio-buccal root; Me, mesial root; MP, mesio-palatal root; p, pulp; PDL, periodontal ligament.

On day 21 of the experiments, the rats were sacrificed by decapitation under ether anesthesia, and the maxillary bilateral 1^st^ molars were carefully extracted by removing the paradental tissues including the soft tissue and alveolar bone around the 1^st^ molars, followed by immersing in 1% sodium hypochlorite for 10 minutes. After the surface of the mesial, mesio- and disto-palatal roots were completely cleared, the mesio- and disto-buccal crown and roots were cut parallel to the midpalatal raphe using dental diamond discs. For standardizing the orientation of the samples, this cut surface was placed on the sample holder, and vertical scanning was performed using a SEM (VE-9800; Keyence, Osaka, Japan). The mesial and mesio- and disto-palatal roots were observed en block from the palatal side under a SEM (Fig. 7a). As described previously^49^, from the obtained images, the root area and area of root resorption lacunae in the 3 roots were measured using software (Image J; National Institutes of Health (NIH)). The root resorption rate for each root (area of root resorption lacunae/root area × 100) was calculated using software (Excel; Microsoft Co., WA, USA).

### Microcomputed Tomography (µCT) analysis for PDL space

On days 7, 9 and 21 of the experiment, perfusion fixation with 4% paraformaldehyde was performed, and the maxillary bone containing the teeth was resected, and fixed with 4% paraformaldehyde for 24 hours and 70% ethanol. Microfocal computed tomography (ScanXmate-E090; Comscan, Kanagawa, Japan) was used in all experimental animal groups in order to clarify the changes in their volume of PDL space (Fig. 7b). The µCT settings were as follows: pixel, 1032 × 1032; pixel size, 0.05 μm; projection, 800; magnification, 6.371; voltage, 88.80 kV; electrical current, 0.107 mA. The TRI/3D-BON64 software (RATOC System Engineering, Tokyo, Japan) was used to generate three-dimensional reconstruction images of the maxilla (Fig. 7b).

### Histomorphometric analysis

On day 9 of the experiments, rats received perfusion fixation with 4% paraformaldehyde, and the maxillary bone containing the teeth was resected, decalcified with 20% ethylenediaminetetraacetate (EDTA), and paraffin-embedded using the routine method. Serial sections (thickness, 5 μm) were prepared. The sections were sliced parallel to the occlusal plane of the upper molars, and the level of the sections from the furcation to the apex was calculated by the counted number of the serially sliced sections^56^. The slides were stored at 4 °C until used for HE staining, TRAP staining, and immunofluorescence.

Histomorphometric analysis was focused on the interradicular septum of the upper first molar. Three sections were evaluated per animal. The first sections were about 415 μm from the furcation of the teeth (Fig. 7c), second ones about 30 μm away, and third ones about 60 μm away. These nine sections per group (n = 3) were used for quantitative analysis. The field surrounded by three lines, which was drawn tangentially to the pulp surface of each root of the upper 1^st^ molar, was the region of interest (ROI) for histomorphometric analysis (Fig. 7d). For measuring the area of osteocyte lacuna, ROI was divided into 6 fields by three median lines of ROI’s triangle (Fig. 7e). Two osteocyte lacunae were selected randomly within each field and totally 12 of osteocyte lacunar areas were measured per section of HE staining.

To identify cell types, we prepared serial sections of HE staining and TRAP staining. TRAP staining was performed with acid phosphatase leukocyte kit (Sigma) according to the manufacture’s instruction. Cells located adjacent to the bone matrix, containing more than 3 nuclei, and positive for TRAP were identified as osteoclasts, and mononuclear cells were as preosteoclasts. Cuboidal cells along bone surface facing periodontal space and marrow space were identified as osteoblasts. Cells with multiple slender cytoplasmic processes located in osseous lacunae were identified as osteocytes. Two-dimensional areas were measured using imaging software ImageJ (NIH). Lengths of perimeters for mesial roots of maxillary 1^st^ molar (bold line in Fig. 7d) were measured with cellSens (OLYMPUS, Tokyo, Japan).

### Immunofluorescence

Sections were deparaffinized in xylene, hydrated, washed three times in PBS, and permeabilized in 0.2% TritonX at room temperature. Then, sections were incubated with anti-NF-κB p65 antibody (c-20; Santa Cruz, CA, USA) of 1:100 dilution at 4 °C overnight. After having been washed with PBS three times, the sections were incubated for 1 hour at room temperature with Alexa Fluor 555 anti-rabbit IgG (Invitrogen, CA, USA). Nuclei were stained with DAPI (KPL, MA, USA). The sections were examined using confocal laser scanning microscopy (C2si; Nikon, Tokyo, Japan) and imaging software NIS-Elements (version 4.13; Nikon). To quantify the areas of NF-κB expression, the fluorescent images obtained from immunostaining were analyzed using imaging software ImageJ (NIH). Thresholds of 80 and 255 were used for definition of NF-κB expression areas in red channel image.

### Statistics

Data of 2-group comparisons were analyzed using Student’s *t*-test. Simultaneous comparisons of more than 2 groups were performed using one-way analysis of variance (ANOVA) followed by *post hoc* analysis by Tukey-Kramer. Differences were considered significant when *P* < 0.05. Data was represented as mean ± SD.
